# Supplementary figures and images for: Ultra-precise quantification of mRNA targets across a broad dynamic range with nanoreactor beads
Source: PLoS One. 2021 Mar 18;16(3):e0242529. doi: 10.1371/journal.pone.0242529 (PMC7971518; doi:10.1371/journal.pone.0242529)

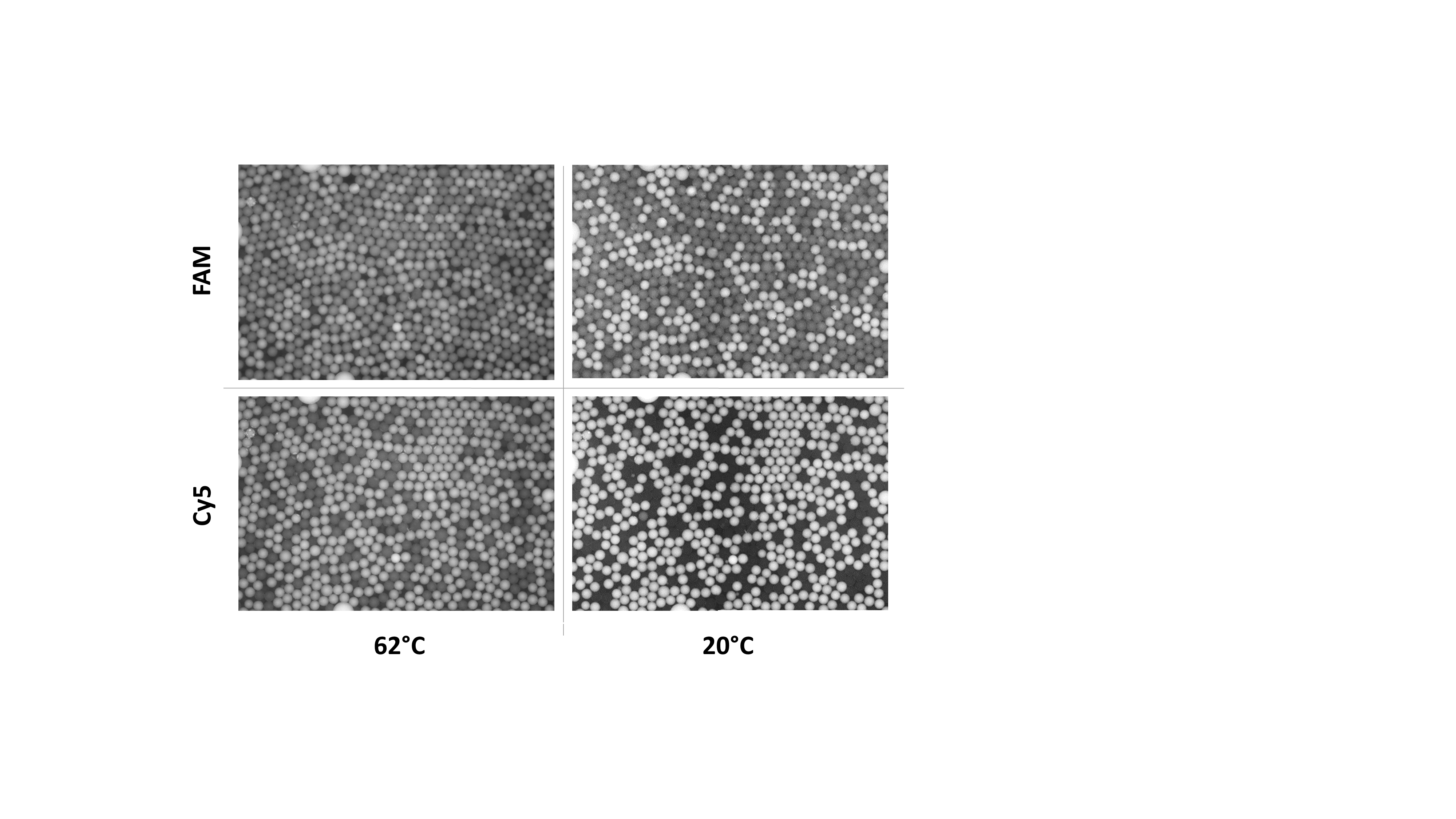

Supplement: S1 Fig — Shown images have been acquired after a PCR run with 40 cycles; FAM, fluorescence of GUSB gene probe; Cy5, fluorescence of BCR-ABL1 gene probe; bright and dark fluorescence represents PCR positive and negative nanoreactor beads, respectively. Positive and negative nanoreactor beads are distinguishable at 62°C and at 20°C. (TIF) [file pone.0242529.s001.tif]

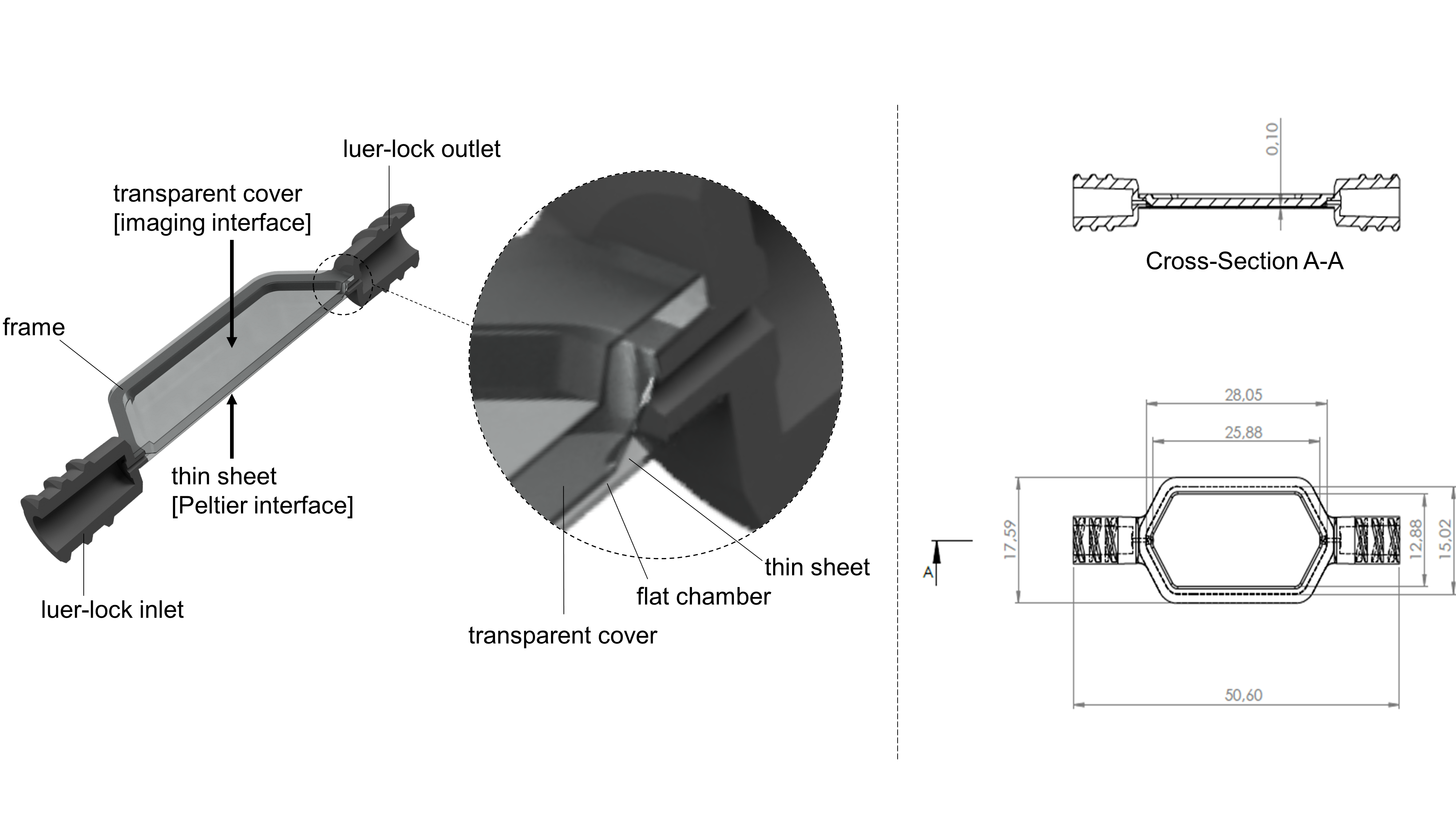

Supplement: S2 Fig — Right, Dimensions of RDC are shown at the right in mm; 0.1 indicates the distance between the transparent cover and the thin sheet. Left, a cross section of a rendered drawing of the RDC. Enlarged inset shows set-up forming a flat chamber for accommodating nanoreactor beads. (TIF) [file pone.0242529.s002.tif]
