# Supplementary material for: Ultra-precise quantification of mRNA targets across a broad dynamic range with nanoreactor beads
Source: PLoS One. 2021 Mar 18;16(3):e0242529. doi: 10.1371/journal.pone.0242529 (PMC7971518; doi:10.1371/journal.pone.0242529)
Supplement: S1 Table — (DOCX) [file pone.0242529.s003.docx]

**S1 Table. Patient samples data.**

| **Sample ID** | **gender** | **Age at diagn.** | **current TKI therapy** | **transcript  type** | **RNA (ng/µl)** | **BCR-ABL1 #1 (abs. cp)** | **BCR-ABL1 #2 (abs. cp)** | **GUSB #1 (abs. cp)** | **GUSB #2 (abs. cp)** |
| --- | --- | --- | --- | --- | --- | --- | --- | --- | --- |
| UKJ42 | f | 50 | asciminib | b3a2 | 230 | 252332 | 247478 | 1074477 | 1080888 |
| UKJ48 | m | 45 | dasatinib | b3a2 | 235 | 228346 | 207988 | 2424183 | 2293056 |
| UKJ52 | m | 52 | dasatinib | b2a2 | 233 | 64626 | 65127 | 945537 | 911289 |
| UKJ41 | f | 47 | asciminib | b3a2 | 168 | 4725 | 3757 | 1361359 | 1464338 |
| UKJ45 | m | 60 | none | b2a2 | 152 | 4273 | 4253 | 1268773 | 1184874 |
| UKJ63 | m | 46 | dasatinib | b3a2 | 100 | 2130 | 2085 | 1051903 | 1029873 |
| UKJ38 | f | 33 | dasatinib | b2a2 | 170 | 1684 | 1734 | 1003401 | 978620 |
| UKJ51 | m | 63 | dasatinib | b3a2 | 122 | 880 | 809 | 746152 | 740736 |
| UKJ60 | m | 78 | ponatinib | b3a2 | 123 | 576 | 566 | 1541809 | 1339202 |
| UKJ62 | m | 64 | nilotinib | b2a2 | 161 | 293 | 241 | 1113915 | 1664090 |
| UKJ67 | m | 64 | dasatinib | b2a2 | 108 | 278 | 265 | 983399 | 1002491 |
| UKJ40 | m | 47 | asciminib | b2a2 | 139 | 100 | 103 | 1552467 | 1486011 |
| UKJ57 | m | 61 | none | b3a2 | 103 | 80 | 69 | 825584 | 915342 |
| UKJ69 | f | 21 | bosutinib | b3a2 | 133 | 42 | 45 | 925010 | 874135 |
| UKJ54 | m | 42 | none | b2a2 | 156 | 23 | 21 | 576296 | 687880 |
| UKJ68 | m | 32 | nilotinib | b3a2, b2a2 | 75 | 19 | 18 | 628069 | 632200 |
| UKJ43 | f | 46 | none | b3a2 | 199 | 17 | 21 | 879792 | 911959 |
| UKJ66 | m | 56 | nilotinib | b3a2 | 66 | 14 | 21 | 826619 | 747621 |
| UKJ64 | m | 49 | none | b3a2 | 63 | 12 | 11 | 397522 | 401582 |
| UKJ58 | m | 66 | none | b3a2, b2a2 | 90 | 8 | 9 | 962658 | 903388 |
| UKJ50 | f | 62 | none | b3a2 | 240 | 7 | 0 | 519886 | 523291 |
| UKJ65 | m | 44 | nilotinib | b3a2 | 88 | 6 | 0 | 284674 | 294016 |
| UKJ47 | m | 46 | none | b3a2 | 146 | 4 | 2 | 1016261 | 1017743 |
| UKJ44 | f | 46 | none | b3a2 | 262 | 0 | 0 | 3033061 | 2920075 |
| UKJ49 | m | 42 | none | b2a2 | 123 | 0 | 0 | 656933 | 600971 |
| UKJ53 | m | 70 | none | b3a2 | 153 | 0 | 0 | 669567 | 817239 |
| UKJ59 | m | 54 | none | b3a2, b2a2 | 137 | 0 | 0 | 1326009 | 1277912 |
| UKJ61 | f | 47 | nilotinib | b3a2, b2a2 | 92 | 0 | 0 | 730803 | 659187 |

UKJ, Sample IDs of the molecular-oncology laboratory at Jena University Hospital; f, female; m, male; TKI, Tyrosine Kinase Inhibitor; abs cp, absolute copies; 1#, replicate 1; 2#, replicate 2.
