# Supplementary material for: Ultra-precise quantification of mRNA targets across a broad dynamic range with nanoreactor beads
Source: PLoS One. 2021 Mar 18;16(3):e0242529. doi: 10.1371/journal.pone.0242529 (PMC7971518; doi:10.1371/journal.pone.0242529)
Supplement: Attachment — Submitted filename: Response to Riviewers.docx [file pone.0242529.s004.docx]

**Response to Reviewer 1**

Overall, the manuscript is well written. The following are some suggestions for the authors to consider for a revised manuscript.

Major:
Since this is mainly an assay development manuscript, the data on assay quality assessment are somewhat insufficient. Only the linear dynamic range is provided. What about assay specificity? Intra-day vs. inter-day reproducibility? Is there any optimization data on beads: samples ration? How about the LLOQ of this assay?

- *In this manuscript we are demonstrating the feasibility of a novel approach for setting up molecular assays with broad dynamic range and high precision based on combined digital and real time readout of fluorescence generated within nanoreactors beads. Since we are presenting basic technical feasibility the clinical application is to be considered only an exemplary model for the kind of assays that would benefit from the new technique. Of course, for use in a clinical context further validation is required.

  Therefore for this initial feasibility assessment we would like to limit the scope of assay characterization to a qualitative assessment of the achieved precision shown by the intraday SD with 6 replicates. The measuring range and linearity by the comparison to the reference laboratory data provides an initial data set for achievable specificity with this assay format.*

Minor:
All the texts in every figures are fuzzy and cannot to be recognized.

- *All images have now been set to 300dpi.*

**Response to Reviewer 2**

The authors present a way to compartmentalize biomolecules using hydrogel beads. They explain very well why this is important in the introduction to achieve true quantitative measurements of nucleic acids as current systems having the q in their name are not really quantitative. This is a very important issue as the COVID19 pandemia have demonstrated. The manuscript is technically sound and well structured. I must say I enjoyed reviewing this manuscript. Hence, I do recommend its publication with some minor revisions. Below, there are a number of recommendations and questions that I'd like the authors to address.

I suggest authors to add a sentence in the introduction to describe the differences between their method and the ones in ref 7 and 8 of their manuscript as this is an important justification of their work.

- *A sentence is now added in line 31- 34 clearly outlining the difference between the referenced technologies and the new method presented in this manuscript.*

Regarding the cell-based assays authors should refer how cells were cultured and also number of cells used. These number of cells could be used to translate to the number of transcript per cell detected. The number of a transcript per cell is always difficult to determine hence this technology could be useful to do so.

- *HL60 cells and K562 cells served only as source for extraction of RNA negative and positive for BCR-ABL1, respectively. Both RNA extracts where used to generate contrived samples with low, middle and high BCR-ABL1/GUSB ratios. Though the presented method indeed could be applied to quantitate transcripts per cell, in this work the actual recovery of RNA-copies per cell did not play any role. We have now clarified this in the text.*

One aspect which is not clear to me is the melting and reforming step of the process. In Fig. 1 (iv.) is said "bead melting, PCR and fluorescence detection". As the reading is done with beads, how long does it take to reform the beads? Are beads melted during the whole PCR process? So, I suggest to explain this step with more detail and add pictures of the 3 min denaturation step either in the main text or supplementary information.

- This is now better explained in the text (line 42-71)

When a sample gives #dark beads <0.5%, it is then quantified by Ct and calibration curve. If that sample is diluted to have a #dark beads >0.5%, it is quantified by Poisson analysis. Are the quantifications equal, taking into account the dilution factor? Fig. 5 might answer it but it is not clear to me if that samples are serial dilutions or not.
Do you need to do a calibration curve per run?

- *A calibration curve is required for the real-time analysis part of the assay, this is if less than 0.5% present in the imaged area remain dark. If the number is >0.5% absolute quantification based on Poisson analysis is feasible. We have added a clarifying statement to the data analysis section.*
- *Adjustment of the low, middle, and high level of BCR-ABL1/ratio is not done by serial dilution. We have mixed extracted RNA from different cell lines at different ratios to order to obtain three distinct levels of BCR-ABL1/GUSB ratios within 6 orders of magnitude.*

Duration of each step in the workflow scheme could be useful for the reader to understand how long the sample-to-result takes.

- *The duration of the test from sample to result is added (L188-L190)*

More details of the commercial suppliers of reagents are needed. It might be good to have a General section at the beginning of the Material and Methods section containing these details.
TaqMan probes references are not provided.
While authors give a reference for primers and probes, a Table describing them would be very helpful for readers

- *A table including all sequences for primers and probes has been added to the text. Wherever appropriate additional information on the source for the used materials has been provided.*

Fig. 1. A scale bar is missing

- *added*

Line 247 there is a reference error

- *corrected*

**Response to Reviewer 3**

The authors report the development of a 'nanoreactor bead' based assay for the quantification of mRNA targets. It appears that the use of nanoreactor beads can be used in quantification of mRNA targets spanning six order of magnitude.

The authors build their manuscript on an argument by stating that digital assays are complex and require sophisticated microfluidic tools. Unless I am missing some important aspects/details the authors do generate 'nanoreactor beads' using a microfluidics chip, and the proposed method by all means not an unsophisticated one. Hence it is not clear to me why they prefer to 'sell' their method to be microfluidics-free and 'easy'.

- *The presented assay format is based on the use of hydrogel beads. These beads are made in bulk in large quantities and stored until they are needed. Contrary conventional droplet PCR requires microfluidic tools and specialized equipment to generate mono-disperse droplet nano-compartments to run the actual test, whereas in the presented format the beads in combination with a fluorocarbon oil and a suitable emulsifier are sufficient for partitioning of the sample into sub-nanoliter sized digital reaction compartments.
  We have now made this clear in the text.*

The manuscript text appears to be not submission ready given the overwhelming number of points I could comment on. The text could benefit from a thorough editing, especially paying attention to inconsistent terminology use that confused me on multiple occasions, especially the nanoreactor, digital nanoreactors, and alike… -

- *Thank you for this input: We have checked the text for consistency with regards to the terminology -
  - nanoreactor beads is now the only term used for the beads
  - Reaction and detection chamber us used throughout the text*

There is also no statistical analysis section provided within the Materials and Methods section.

- *A statistical analysis section has been added to the Materials and Methods*

And finally the method section can benefit from a detail oriented focus.
Given the current status of the manuscript, I would conclude resubmission after major revision; however, in this journal it corresponds to major revision hence my decision. Below I list my various comments based on the page and line numbers:

Cover page: there are typos in the keywords!

- *Corrected*

Page 2, Line 16: good agreement -> Can the authors use a quantitative measure. Also while the title reads 'ultra-precise' the closing statement of the abstract states 'good agreement'. It is ultra-precise or good? (I personally dislike such use of prepositions. [What would be the next stage? Ultra²?])

- *Level of concordance has been quantified by Pearsons correlation coefficient of 0.983 and slope of 1.08.*

P3, L23: partitions -> It is not clear what is meant by partition. Can the authors clarify (the figure 1 legend reads beads. Can you please tune the term use for consistency/clarity).

- *We have added a description what partition means in line 23.*

*In this manuscript we are referring to the basic concepts of digital PCR whereof sample partitioning is the fundamental approach. The sample is divided into a number of identical sub-volumes. A partition represents such a unit sub-volume. Since Fig 1 is referring to the newly developed process the figure capture refers to “nanoreactor beads”, whereas the introductory sentences are referring to digital analysis in general, hence our use of “partitions” there.*

P3, L24: Poisson criteria -> Poisson distribution (criteria?). Can you please clarify what the criteria is/are?

- *The unclear statement has been removed. The underlying mathematical model is explained in the quoted reference [5].*

P3, L29: digital compartments -> for consistent terminology use can you please stick to one term whether it is the digital compartments/beads/nanoreactor compartment/nanoreactor beads/microbeads/sub-nanoliter droplets (whichever term the authors prefer so long as it is used consistently throughout the manuscript.)

- *Thank you, this has been addressed*

P3, L29: generating digital compartments -> Can you present how your droplet generation does not necessitate microfluidics as compared to the standard. Also lay out the details of the concrete differences between the two techniques for droplet generation.

- *A clarifying sentence has been added (P3, L31-34)*

P3, L35: 'amplification reagents and the extracted nucleic acid.' -> 'RT-PCR amplification reagents and the extracted nucleic acids (GUS8 and BCR-ABL RNA).'
Also the text reads BCR-ABL1 RNA. Please stick to one term.

- *We have checked the text for consistency with regards to the terminology.*

P3, L 40: Please state the size of the beads.

- *Scale bar and information on size and volume of the beads has been added to the figure caption*

Figure 1. In panel a please add sub-sections i., ii, … so that the figure complies with the figure legend.

- *The changes have been made to Fig 1*

Figure 1b and c. Can you provide the scale.

- *Scale bars have been added to the image*

P4, L47: extensive shaking -> Can you provide reproducible quantitative information.

- *The process is now described in more detail in line 167-171 in the materials and methods section.*

Figure 2 legend:
Can you please use the same terms both in the figure and the legend, i.e. Fluorescence Micro-imager vs epi-fluorescence microscope, BLINK vs Blink… the text was checked for consistency
. Can you provide the technical details of the chamber i.e. material, dimensions, thickness, alike… in the legend. Please state the company and also provide a link to the software/website.

- *Information has been added to figure legend*

P5, L69: nanoreactor compartment -> see comment P3, L29

- *The sentence has been modified for clarity*

P5, L70: modest number -> Please provide quantitative information wherever possible.

- *The quantity has been added.*

P5, L71: highly precise -> Can the authors avoid the use of qualitative adverb and state the quantitative findings.

- *We have modified the sentence.*

P5, L89: fluidic connection between tubing and chip -> Please provide a link to the chip or provide the design in supplementary.

- *the described set up used for generating nanoreactor beads represents a variant of the commercially available µEncapsulator system. The setup and components are described in detail in the product literature of Dolomite microfluidic. The manufacturer is referenced accordingly.*

P5, L99: was removed -> was removed by?

- *Sentence has been corrected*

P6, L112: e13a2 or e14a2 BCR-ABL1 -> Please describe what e13a2 and e14a2 are.

- *we have changed the names of the transcripts to the more common and known nomenclature (b2a2 and b3a2) and added a short description.*

These are transcript variants that are typical for the CML phenotype. A possible differential impact of the respective transcript variants e13a2 ("b2a2") and e14a2 ("b3a2") on disease phenotype and outcome is still a subject of debate.

- *We confined the analysis to samples with the common genotypes in order to focus on the technology rather than the disease. Thus, we also added a statement to the sample selection and sample exclusion strategy in section samples.*

P7, L113: BCR-ABL-1 -> BCR-ABL1 OK

- *Now consistent BCR-ABL1.*

P7, L119: HL60 and K562 -> Please describe what are HL60 and K562.

- *A description and references are added (L166-L167).*

P7, L124: as described elsewhere -> Please provide all the necessary details in the methods section.

- *A table is added including all sequences for primers and probes*

P8, L143: latter -> There is a single removal step in the previous sentence. Please re-phrase.

- *OK*

P8, L148: parameters -> Please provide the full details.

- *all necessary information is provided in the subsequent sentence.*

Figure 3 legend: Please describe the different beads in the legend. Preferably arrows can also be used to highlighting the specific beads.

- *Arrows have been added to the images for better understanding and the legend modified, accordingly*

P11, L208: ERM-AD623 -> What is ERM-AD623? Please describe.

- *ERM-AD623 is described in L172-175*

P11, L209: (data not shown) -> Please provide the necessary data.

- *Removed, as not necessary in this context*

P11, L211: high copy numbers -> high copy numbers (of what?)

- *Sentence has been corrected*

P11, L213: The concentration levels represent the expected measuring range for clin(ic)al samples -> Please provide data or a reference to support this statement.

- *References supporting the statement have been included*

Figure 5: BCRLAB -> BCR-ABL1.
Also please use different colours for dots and triangles.

- *Images have been modified*

P13, L247: Table 1. Error! Reference source not found. -> Cross reference error?

- *Corrected*

P14, L260; L267: The molecular response (MR) -> Acronym was already introduced in line 258.

- *Corrected*

P14, L267: considered -> Please provide a reference for this statement.

- *References supporting the statement have been included*

P14, L273: precision -> Please provide a reference for this statement.

- *References supporting the statement have been included*

P15, L285: 53.000 -> 53,000?

- *Corrected*

P15, L294: Blink test -> What is a Blink test?

- *Replaced with “new test”*

P16, L303: the reference method -> Please help the reader by providing a legend that provides all the relevant information.

- *Reference method has been explained in line 139 - 140*

P16, L307: same data -> which data? Fig. 7

- *Rephrased and information competed (Fig 8., L512)*

P16, L318: microfluidics free digital molecular assays -> Unless I am missing the picture, the nanoreactor beads were generated using a Dolomite microfluidics chip. So how can the authors claim that the process is microfluidics free?

- *Our statement is correct, as no specific microfluidic tools are required to split the sample into digital partitions during the test, instead we use pre-made hydrogel beads off the shelf for partitioning the targets into digital compartments.*

P16, L320: bead-nanoreactors -> is this the same as nanoreactor beads?

- *Corrected for consistency*

P16, L324: for the ratios -> for the ratios of

- *corrected*

P16, L329: current laboratory standard -> which standard?

- *Rephrased for clarity*
